# In vivo evaluation of safety and performance of a tapered nitinol venous stent with inclined proximal end in an ovine iliac venous model

**DOI:** 10.1038/s41598-024-58237-x

**Published:** 2024-04-01

**Authors:** Zhongjian Wu, Zhengtong Zhou, Chunjing Bian, Lianrui Guo, Zhu Tong, Jianming Guo, Lixing Qi, Shijun Cui, Chengchao Zhang, Yilong Chen, Wei Huang, Yongquan Gu

**Affiliations:** 1 Department of Vascular Surgery, Xuanwu Hospital, Capital Medical University, 45 Changchun Street, Xicheng District, Beijing, 100053 China; 2 Vascular Surgery, The First Affiliated Hospital of Shandong First Medical University, Jinan, China; 3 General Surgery, Xuanwu Hospital, Capital Medical University, Beijing, China; 4 ShenZhen KYD Biomedical Technology Co. Ltd, Guangzhou, China

**Keywords:** Iliac vein, Iliocaval venous obstruction, Venous insufficiency, Venous stent, Biochemistry, Biophysics

## Abstract

A tapered stent with inclined proximal end is designed for fitting the iliac anatomically. The aim of the present study was to evaluate the safety and performance of the new stent in ovine left iliac veins. The experiment was performed in 30 adult sheep, and one nitinol-based VENA-BT^®^ iliac venous stent (KYD stent) was implanted into each animal’s left common iliac vein. Follow-up in all sheep consisted of angiographic, macroscopic, and microscopic examinations at Day 0 (< 24 h), Day 30, Day 90, Day 180 and Day 360 post-stenting (six animals per each time-point). 30 healthy ~ 50 kg sheep were included in this study and randomly divided into five groups according to the follow-up timepoint. All stents were implanted successfully into the left ovine common iliac vein. No significant migration occurred at follow-up. There is no statistically significant difference between the groups (*p* > 0.05), indicating no serious lumen loss occurred during the follow-up period. Common iliac venous pressure was further measured and the results further indicated the lumen patency at follow-up. Histological examinations indicated that no vessel injury and wall rupture, stent damage, and luminal thrombus occurred. There was moderate inflammatory cell infiltration around the stent in Day-0 and Day-30 groups with the average inflammation score of 2.278 and 2.167, respectively. The inflammatory reaction was significantly reduced in Day-90, Day-180 and Day-360 groups and the average inflammation scores were 0.9444 (*p* < 0.001, Day-90 vs Day-0), 1.167 (*p* < 0.001, Day-180 vs Day-0) and 0.667 (*p* < 0.001, Day-90 vs Day-0), respectively. The microscopic examinations found that the stents were well covered by endothelial cells in all follow-up time points. The results suggested that the KYD stent is feasible and safe in animal model. Future clinical studies may be required to further evaluate its safety and efficacy.

## Introduction

The occurrence of left common iliac obstruction secondary to compression of the left iliac vein by the right common iliac artery is called iliac vein compression syndrome (IVCS), about 22% of the population bear this underlying anatomic variant, In patients with IVCS, venous reflux, venous valve insufficiency, varicosities and chronic venous insufficiency will occur because of the long-term compression of the iliac vein^1–5^. More importantly, IVCS is considered as the most common secondary risk factor for deep vein thrombosis (DVT), which can lead to deadly pulmonary embolism (PE).

Stent implantation technology can relieve the obstruction of the outflow tract, and can significantly improve the reflux of the deep veins and protect the valve function. The key to the smooth implementation of this technology is adequate iliac vein opening and accurate positioning as well as release of the stent. Compared with the stent for arterial angioplasty, the stent of deep venous system needs higher flexibility, larger diameter, and higher radial force to overcome the anatomical challenges and high elastic recoil properties of veins^6^. Although a few kinds of stent have been used in the iliac vein, but none of them is fully matched the anatomical challenges. For fitting the human iliac vein anatomically, a tapered stent with inclined proximal end is designed (part of it is cylindrical stent) (VENA-BT^®^ iliac venous stent, ShenZhen KYD Biomedical Technology Co. Ltd., China), which is a nitinol-based self-expanding cone-shaped venous stent with a bevel-shaped proximal end and a distal flat end. The aim of the present study was to evaluate the safety and performance of KYD stent in an ovine model.

## Materials and methods

In accordance with the federal animal protection law, this study received approval of the ethics Committee of Xuanwu Hospital, Capital Medical University. We confirm that all methods were performed in accordance with the relevant guidelines and regulations.

### Animal model

Due to similar anatomy, physiology and coagulation system to human being^7^, sheep was selected as the animal model of this study. The average diameter of the left iliac vein of sheep is 10.06 mm, close to the diameter of the left common iliac vein of human being (~ 15.7 mm)^1^. 30 healthy ~ 50 kg sheep were randomly divided into five groups: Day-0 (< 24 h), Day-30, Day-90, Day-180 and Day-360 groups according to the follow-up timepoint. The sample size in each group (timepoint) is consistent with the previous studies by using sheep as the animal model to evaluate the iliac artery or vein stent^8,9^. Detailed information of the sheep in each group were shown in Table 1.

**Table 1:** Detailed animal information.

Group No.	Serial No.	Animal ID	Weight (kg)	Date of stenting	Date of euthanization	Days of stenting
D-0	1	S097	43.5	09/20/2019	09/20/2019	0
	2	S098	47.5			
	3	S099	45			
	4	S100	48			
	5	S101	46			
	6	S102	43.8			
D-30	1	S088	50	09/11/2019	10/12/2019	30
	2	S089	46			
	3	S090	42			
	4	S093	45			
	5	S095	55			
	6	S096	48			
D-90	1	S079	42	09/02/2019	12/02/2019	90
	2	S080	42.5			
	3	S081	49			
	4	S082	40			
	5	S083	42.5			
	6	S084	60			
D-180	1	S052	45	08/24/2019	04/04/2020	221
	2	S055	48			
	3	S056	42			
	4	S057	41			
	5	S059	43			
	6	S060	35			
D-360	1	S134	38	09/11/2019	09/04/2020	358
	2	S091	49	12/02/2019	12/11/2020	374
	3	S169	50	05/23/2020	05/27/2021	370
	4	S170	52			
	5	S171	51			
	6	S172	50	05/23/2020	05/25/2021	368

### Bliding

During the implementation phase of the experiment, only the experimental personnel were informed of the group assignments, with others being kept uninformed.

### Stent system

**Figure 1:**
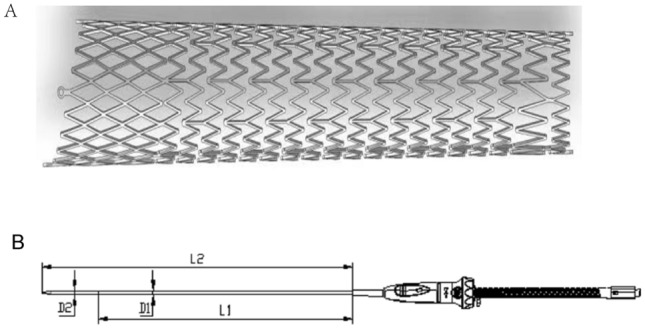
Keyidun stent system. (**A)** The structure of the Keyidun iliac vein stent. (**B**) Schematic diagram of the structure of Keyidun’s iliac vein stent delivery device.

KYD stent system, specifically for fitting the iliac anatomically, was designed by ShenZhen KYD Biomedical Technology Co. Ltd., China. This laser-cut and self-expandable stent is made of nickel-titanium alloy with a beveled proximal end, the proximal part of the stent is the closed-cell stent, while the distal part is the open-cell stent. The two parts are soldered together into the smoothly tapered stent. This specific design ensures the radial support of the stent as well as enough flexibility. (Fig. 1A). The nickel-titanium alloy used in the stent is antimagnetic, thus not interfering with MRI. If the stents were made by magnetic material, the displacement of stents could occur during the MRI scan, which might cause serious injury to human body. Thus, antimagnetic properties of the stents are essential for the safety. KYD Stents with different diameters at both ends are available (part of it is cylindrical stent), which is designed with high crush resistance, flexible interconnections specifically for use in the central venous (Table 2). The stent with the same proximal and distal diameter, that is cylindrical stent, was prepared for some special cases, e.g. similar proximal and distal diameter of the iliac veins. The stent is pre-installed in the sheath of the delivery device. And the delivery device consists of a handle assembly, a push spring tube assembly, a PI (polyimide) sheath core tube (with tip tip), a delivery sheath tube, an outer sheath tube, and a 3-way valve assembly (Fig. 1B). The specifications and dimensions of each stent delivery device are shown in Table 3.

**Table 2:** Specifications and parameters of KYD stent.

Specification	Proximal diameter D1 (mm)	Distal diameter D2 (mm)	Length L (mm)	Conveyor specifications
1010060/1010080/1010100/1010120	10	10	60/80/100/120	9.5Fr (3.20 mm)
1210060/1210080/1210100/1210120	12	10	60/80/100/120	10.5Fr (3.55 mm)
1212060/1212080/1212100/1212120	12	12	60/80/100/120	10.5Fr (3.55 mm)
1410060/1410080/1410100/1410120	14	10	60/80/100/120	10.5Fr (3.55 mm)
1412060/1412080/1412100/1412120	14	12	60/80/100/120	10.5Fr (3.55 mm)
1610060/1610,080/1610100/1610120	16	10	60/80/100/120	10.5Fr (3.55 mm)
1612060/1612080/1612100/1612120	16	12	60/80/100/120	10.5Fr (3.55 mm)
1810060/1810080/1810100/1810120	18	10	60/80/100/120	11Fr (3.7 mm)
1812060/1812080/1812100/1812120	18	12	60/80/100/120	11Fr (3.7 mm)

**Table 3:** Iliac vein stent delivery device specifications from KYD.

Specification model	Sheath cannula length L1 (mm)	Outer diameter of Sheath casing D1 (mm)	Actual length of sheath used L2 (mm)	Outer diameter of delivery sheath D2 (mm)
9.5F	240	3.4	400	3.2
10.5F	240	3.75	400	3.55
11F	240	3.9	400	3.7

### Stenting procedure

After fasting for 12 h, the sheep was anaesthetized and fixed on the operating table in the supine position. After tracheal intubation, the sheep was ventilated with O_2_/NO_2_. The anesthesia was maintained under continuous intravenous infusion of anesthetics, and vital signs were monitored. After skin preparation and disinfection, puncture the left femoral vein, insert a 6F catheter sheath, and inject 100 U/kg heparin through the catheter sheath. A 5F pigtail catheter was sent to the iliac vein through the catheter sheath, and the bilateral iliac veins and vena cava were examined by angiography. The diameter and length of the left iliac vein of each sheep were recorded. The iliac vein stent was introduced and released under DSA. The proximal end of the stent would be deployed at the confluence of the iliac veins with no extension into the IVC.

Postoperative angiography was performed again to confirm the shape and position of the stent, and the puncture point was compressed to stop bleeding.

### Postoperative management

4.8 million units of penicillin were injected intramuscularly within 24 h after the operation, and then 4.8 million units of penicillin were injected intramuscularly every day for 3 consecutive days. After the animals regained consciousness, they were fed and observed for 1, 3, and 6 months. Each sheep was given 100 mg of oral aspirin and 75 mg of clopidogrel per day for 3 months after surgery, and then 100 mg of oral aspirin per day until being euthanized. The efficacy of aspirin and clopidogrel in sheep seems controversial^10,11^. But antiplatelet treatment in sheep post stenting were still performed in some recent studies^9,12^. Thus, aspirin and clopidogrel were also given to the sheep in this study, with the same of dosage of that in stented patients. After indicated follow-up time, the animals were euthanized by intravascular injection of heparin and overdose of anesthetic.

### Study outcome measures

Stenting success was defined as deployment of the stent within 5 mm of the intended location in the iliac system. Stent fracture was examined by digital subtraction angiography (DSA). The degree of Stenting oversizing relative to vein size was evaluated by calculating the compression ratio. For evaluating the acute and long-term lumen patency, lumen loss rate was calculated and common iliac venous pressure (CIVP) was measured.

#### Compression ratio calculation

According to the proximal diameter and distal diameter of iliac vein measured by angiography before iliac vein implantation (Div), and the diameter of the implanted stent (Ds), the compression ratio of stent was calculated with the following formula.

$${\text{Compression ratio }}\left( \% \right)\, = \,\frac{Ds - Div}{{Ds}} \times 100$$

#### Lumen loss evaluation

Angiographic reviews were performed at day 0 (< 24 h), Day 30, Day 90, Day 180, and Day 360 post-stenting. The minimum lumen diameter (MLD) of the iliac vein stent was measured from the venography images immediately after the procedure and at each follow-up endpoint, and the lumen loss (in mm) and lumen loss rate were calculated according to the following formulas respectively:

$${\text{Lumen loss}}\, = \,{\text{iliac vein stent MLD in the immediate postoperative period}} - {\text{iliac vein stent MLD at the follow - up endpoint}}.$$

$${\text{Lumen loss rate }}\left( \% \right)\, = \frac{{\text{immediate postoperative MLD - follow - upMLD }}}{{\text{immediate postoperative MLD}}}\, \times 100\%$$

#### CVIP measurement

The BeneView T5 monitor was used to measure CVIP. The zero point was set when water level in calibration catheter was on the level of the sheep’s right atrium. After successful puncture, the distal end of the pigtail catheter was placed at the confluence of the iliac veins. All air was removed from the lumen of the catheter, and then it was connected to the manometry catheter to measure the pressure of proximal CVIP. Subsequently, the pigtail catheter was removed, and the flushing catheter of the puncture sheath was connected to manometry catheter after complete air removal to measure the pressure of distal common iliac vein. The procedure was performed before and immediately after stenting, as well as at each follow-up timepoint.

### Histologic examination

Three parts of the stented veins (distal, middle, and proximal/closest to the IVC) were examined histologically with a total of 60 sections. Hematoxylin and eosin staining (H&E staining) of stented vein section were performed and examined under light microscopy. Morphometric computer-assisted methods and software was used for image capture and quantitative analysis.

### Inflammation score

Parastrut inflammation was defined as the presence of macrophages and foreign body giant cells admixed with variable numbers of lymphocytes. The inflammation score was calculated according to the following criteria (Table 4). For each timepoint per animal, the leukocytes were counted in totally 30 high-powered fields to calculate the inflammation score, 10 from the histological section of the distal end of the stent, 10 from the one of the middle part of stent, and 10 from the one of the proximal end of the stent, respectively. Thus, for six animals in each time point per group, the leukocytes was counted in totally 180 HP fields and the inflammation score was calculated.

**Table 4:** Inflammation scoring criteria.

Scoring	Specifications
0	Leukocytes < 5/HP
1	5 < leukocytes < 10/HP
2	10 < leukocytes < 20/HP
3	Leukocytes > 20 cells/HP

*HP* high-power field.

#### Endothelialization examination

The cross sections of the stented veins at indicated follow-up timepoint were evaluated under high-powered microscopy after hematoxylin and eosin staining to evaluate for the presence of a layer of endothelial cells covering the lumen.

### Statistical analysis

The data were analyzed using SPSS software (version 20, SPSS Inc., USA). Statistical differences between groups were compared using ANOVA and *t*-test. For the inflammation score, Kruskal–Wallis test was used to analyze the statistical difference between the groups. *p* < 0.05 was considered statistically significant if not otherwise specified.

### Statement

The study is reported in accordance with ARRIVE guidelines.

## Results

### Stent insertion and migration

**Figure 2:**
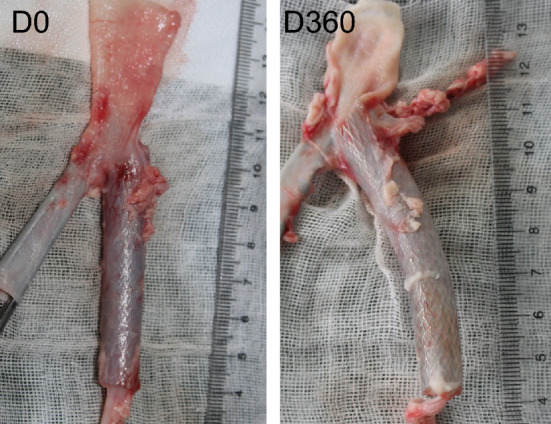
Dissection of the stented segment of the iliac vein at indicated timepoint.

30 sheep were successfully implanted with iliac vein stents in the left common iliac vein via the left femoral vein under DSA guidance. The release of the bracket is successful, and the positioning of the bracket is accurate (Fig. 2).

**Figure 3:**
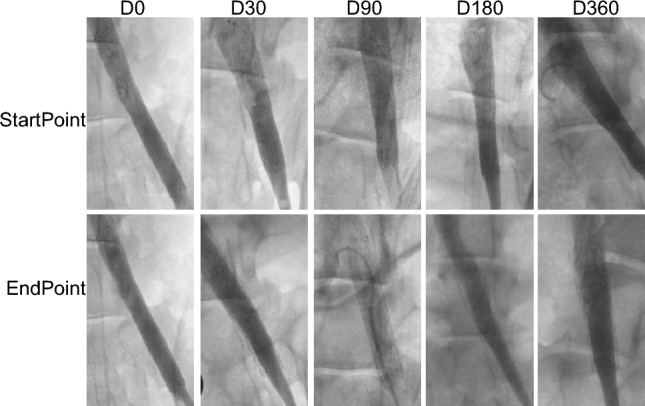
Angiograms of immediate poststent (< 24 h) and each follow-up timepoint. StartPoint: angiography was performed within 24 h post stenting; EndPoint: angiography was performed at indicated follow-up timepoint.

In all the 30 sheep, no damage of the iliac vein or stent fracture was observed by angiography immediately after implantation of the iliac vein stent (Fig. 3).

During follow-up, no stent displacement, stent fracture or stent placement-related complications, such as bleeding, vein injury and thrombosis, were observed by DSA angiography in all groups. The positioning of the proximal end of the stent is accurate, and the imaging re-examination shows that the position of the proximal end of the stent is the same as before, without displacement, and no shortening is observed. (Fig. 3).

### Stent compression ratio

**Figure 4:**
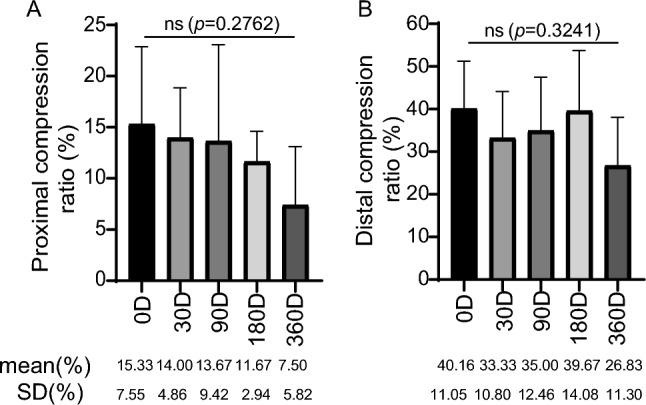
Stent compression ratio of proximal and distal end for each group. Data are shown as mean ± SD. *ns* non statistical significance.

The selection of stent diameter in this study was based on the compression ratio (10–30%)^12^. The minimum proximal compression ratio of the deployed stent was 3.3%, the maximum was 30.3%, and the mean was 12.4%. As for the distal compression ratio the minimum was 14%, the maximum was 56.9%, and the mean was 35% (Fig. 4, Table 5). No significant difference was shown in either proximal or distal compression ratio among groups (Fig. 4). 60% (18/30) implanted stents had a distal compression ratio of more than 30%.

**Table 5:** Preoperative angiography and stent compression ratio.

Group No	Serial No	Animal ID	Stent specifications	Diameter of vein (mm)		Diameter of stent (mm)		Stent compression ratio		Mean ± SD	
				Proximal	Remote	Proximal	Remote	Proximal (%)	Remote (%)	Proximal (%)	Remote (%)
D-0	1	S097	1010060	8.89	6.5	10	10	11	35	15.33 ± 7.55	40.16 ± 11.05
	2	S098	1210060	8.36	7.57	12	10	30	24		
	3	S099	1010060	9.07	5.01	10	10	9	50		
	4	S100	1210060	10.2	4.51	12	10	15	55		
	5	S101	1010080	8.46	6.1	10	10	15	39		
	6	S102	1410080	12.3	6.6	14	10	12	38		
D-30	1	S088	1010060	7.85	4.84	10	10	22	52	14.00 ± 4.86	33.33 ± 10.80
	2	S089	1210060	10.9	6.47	12	10	9	35		
	3	S090	1410080	12	7.63	14	10	14	24		
	4	S093	1410060	11.8	7.37	14	10	16	26		
	5	S095	1210060	10.9	6.18	12	10	9	38		
	6	S096	1210080	10.3	7.47	12	10	14	25		
D-90	1	S079	1210060	9.11	6.04	12	10	24	40	13.67 ± 9.42	35.00 ± 12.46
	2	S080	1210060	10.2	8.27	12	10	15	17		
	3	S081	1010080	7.56	6.42	10	10	24	36		
	4	S082	1210060	11.6	4.6	12	10	3	54		
	5	S083	1210080	11.6	7.33	12	10	3	27		
	6	S084	1210060	10.4	6.4	12	10	13	36		
D-180	1	S052	1010060	8.9	6.88	10	10	11	31	11.67 ± 2.94	39.67 ± 14.08
	2	S055	1210060	10.5	7.26	12	10	13	27		
	3	S056	1010060	9.16	7.39	10	10	8	26		
	4	S057	1210080	10.1	4.31	12	10	16	57		
	5	S059	1210060	10.9	4.4	12	10	9	56		
	6	S060	1210060	10.4	5.87	12	10	13	41		
D-360	1	S134	1210060	11.2	7.26	12	10	7	27	7.50 ± 5.82	26.83 ± 11.30
	2	S091	1010060	8.13	5.82	10	10	19	42		
	3	S169	1410060	13.4	6.31	14	10	4	37		
	4	S170	1410080	13.4	8.6	14	10	4	14		
	5	S171	1412060	13.5	10.2	14	12	4	15		
	6	S172	1610100	14.9	7.4	16	10	7	26		

### Lumen loss

**Figure 5:**
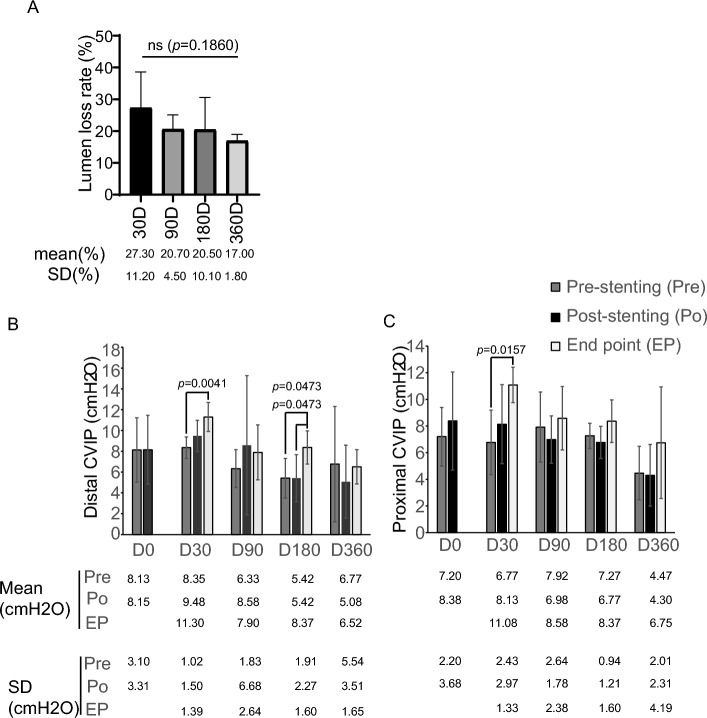
Evaluation of lumen patency. (**A**) Lumen loss rate of stent at each follow-up time-point was calculated according to the angiographic results. (**B**–**C**) Distal and proximal CVIP were measured at each follow-up time-point by using BeneView T5 monitor. Data are shown as mean ± SD. *ns* non statistical significance. *p < 0.05; **p < 0.01.

In present study, the average lumen loss rate was 27.3% (14.1–46.6%) in Day-30 group, 20.7% (15.8–28.0%) in Day-90 group, 20.5% (8.0–31.8%) in Day-180 group and 17.0% (13.5–19.0%) in Day-360 group, respectively (Fig. 5A, Table 6). No significant difference in lumen loss rate was observed among the four follow-up groups (Fig. 5A, Table 6). No lumen loss rate in individual sheep was higher than 50% at all follow- up time points (Fig. 5, Table 6).

**Table 6:** Lumen loss rate.

Group No	Serial No	Animal ID	Stent specifications	Immediate post-stent MLD	Follow-up MLD	Lumen loss (LL) (mm)	Lumen loss rate (%)	Mean ± SD (%)
D-0	1	S097	1010060	8.16	8.16	0	0	0
	2	S098	1210060	8.22	8.22	0	0	
	3	S099	1010060	7.84	7.84	0	0	
	4	S100	1210060	7.44	7.44	0	0	
	5	S101	1010080	8.22	8.22	0	0	
	6	S102	1410080	8.09	8.09	0	0	
	1	S088	1010060	9.81	6.81	3	30.6	27.3 ± 11.2
	2	S089	1210060	8.31	7.14	1.17	14.1	
	3	S090	1410080	10.2	5.45	4.75	46.6	
D-30	4	S093	1410060	8.29	6.57	1.72	20.7	
	5	S095	1210060	8.53	6.64	1.89	22.2	
	6	S096	1210080	9.32	6.54	2.78	29.8	
	1	S079	1210060	8.45	6.7	1.75	20.7	20.7 ± 4.5
	2	S080	1210060	8.74	6.7	2.04	23.3	
	3	S081	1010080	6.93	5.61	1.32	19.0	
D-90	4	S082	1210060	7.74	6.42	1.32	17.1	
	5	S083	1210080	8.97	6.46	2.51	28.0	
	6	S084	1210060	10.1	8.5	1.6	15.8	
	1	S052	1010060	8.33	6.18	2.15	25.8	20.5 ± 10.1
	2	S055	1210060	9.05	6.17	2.88	31.8	
	3	S056	1010060	9.59	8.67	0.92	9.6	
	4	S057	1210080	7.41	5.25	2.16	29.1	
D-180	5	S059	1210060	8.17	6.64	1.53	18.7	
	6	S060	1210060	8.09	7.44	0.65	8.0	
	1	S134	1210060	13.9	11.6	2.3	16.5	17.0 ± 1.8
	2	S091	1010060	7.68	6.37	1.31	17.1	
	3	S169	1410060	15.8	12.8	3	19.0	
D-360	4	S170	1410080	9.25	8	1.25	13.5	
	5	S171	1412060	10.5	8.64	1.86	17.7	
	6	S172	1610100	20.9	14.9	6	18.4	

**Figure 6:**
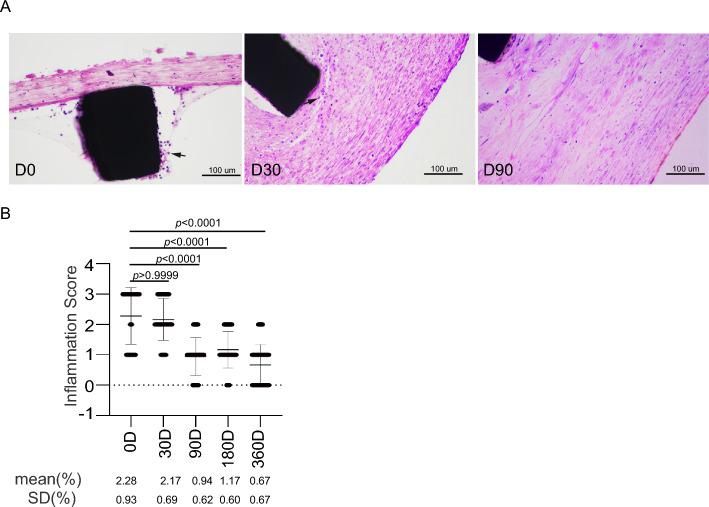
Inflammatory response around stent. (**A**) H&E staining shows inflammatory cell infiltration (black arrow) in the intima at indicated timepoint. (**B**) Inflammation score of each timepoint was calculated according to the H&E staining images. Data are shown as mean ± SD.

To further evaluate the venous patency, CIVP was also measured at follow-up. Vein stenosis may cause the rise of venous pressure. Our results indicated that neither distal nor proximal CIVP showed significant alteration during the acute period of stenting (< 24 h) (Fig. 5B, C and Table 7). In contrast, both distal and proximal CIVP at day 30 post-stenting were slightly increased (Fig. 5B, C and Table 7), which might be related to intima hyperplasia (Fig. 6A). In Day-90 and Day-360 groups, there was no significant change of the final CIVP at day 90 or day 360 compared to pre-stenting CIVP. However, the distal but not proximal CIVP at day 180 after stenting was higher than pre-stenting CIVP. Considering no significant lumen loss was observed in Day-180 groups, the increase of distal CIVP may not be related to alteration of venous patency post stenting. Overall, the KYD stents demonstrated satisfactory performance with regard to the long-term lumen patency.

**Table 7:** Left common iliac venous pressure (cmH_2_O).

Group No	Serial No	Animal ID	Distal		Proximal		Distal	Proximal
			Pre-stent	Post-stent	Pre-stent	Post-stent	End point	
D-0	1	S097	13.6	12.2	8.1	13.6	NA	NA
	2	S098	4.1	4.1	2.7	4.1	NA	NA
	3	S099	6.8	4.1	8.1	4.1	NA	NA
	4	S100	8.1	9.5	8.1	9.5	NA	NA
	5	S101	8.1	9.5	8.1	9.5	NA	NA
	6	S102	8.1	9.5	8.1	9.5	NA	NA
D-30	1	S088	8.1	9.5	2.7	4.1	12.2	10.9
	2	S089	9.5	8.1	5.4	5.4	12.2	12.2
	3	S090	8.1	12.2	6.8	12.2	12.2	12.2
	4	S093	6.8	9.5	8.1	9.5	9.5	9.5
	5	S095	9.5	9.5	9.5	8.1	12.2	12.2
	6	S096	8.1	8.1	8.1	9.5	9.5	9.5
D-90	1	S079	8.1	21.7	9.5	8.1	12.2	12.2
	2	S080	8.1	6.8	10.9	8.1	5.4	5.4
	3	S081	6.8	8.1	9.5	8.1	9.5	9.5
	4	S082	4.1	5.4	5.4	5.4	5.4	9.5
	5	S083	6.8	6.8	8.1	8.1	8.1	8.1
	6	S084	4.1	2.7	4.1	4.1	6.8	6.8
D-180	1	S052	8.1	6.8	8.1	8.1	8.1	6.8
	2	S055	6.8	8.1	6.8	6.8	6.8	6.8
	3	S056	5.4	5.4	6.4	6.8	10.9	10.9
	4	S057	5.4	6.8	8.1	8.1	9.5	9.5
	5	S059	2.7	2.7	8.1	5.4	8.1	8.1
	6	S060	4.1	2.7	6.1	5.4	6.8	8.1
D-360	1	S134	5.4	5.4	5.1	4.1	5.1	4.1
	2	S091	17.6	11.5	6.8	8.1	6.8	6.8
	3	S169	5.4	4.1	5.4	5.4	9.5	9.5
	4	S170	5.4	5.4	5.4	4.1	5.4	5.4
	5	S171	1.4	1.4	1.4	1.4	5.4	1.4
	6	S172	5.4	2.7	2.7	2.7	6.9	13.3

### Histological examination

#### Gross appearance

At each follow-up time point, the iliac vein segment at the stenting site was dissected and exposed. No vascular wall rupture, thrombosis, or stent damage was observed in any of the sheep. No adhesion between the iliac vein and the surrounding tissue occurred (Fig. 2).

#### Inflammatory response

Inflammatory response caused by stent was mostly seen in the Day 0 and Day 30 group. Immediately after stenting, punctate as well as focally infiltrated inflammatory cells with inflammatory exudate were seen around the stent, and more scattered inflammatory cells infiltration was presented in the intima at day 30 post procedure (Fig. 6A). After 90 days of stenting, inflammatory response was markedly alleviated (Fig. 6A). To better evaluate the inflammatory response, the inflammation score was calculated. Significant difference was observed between Day-0 and Day-90/Day-180/Day-360 (Fig. 6B).

#### Intima hyperplasia and endothelialization

**Figure 7:**
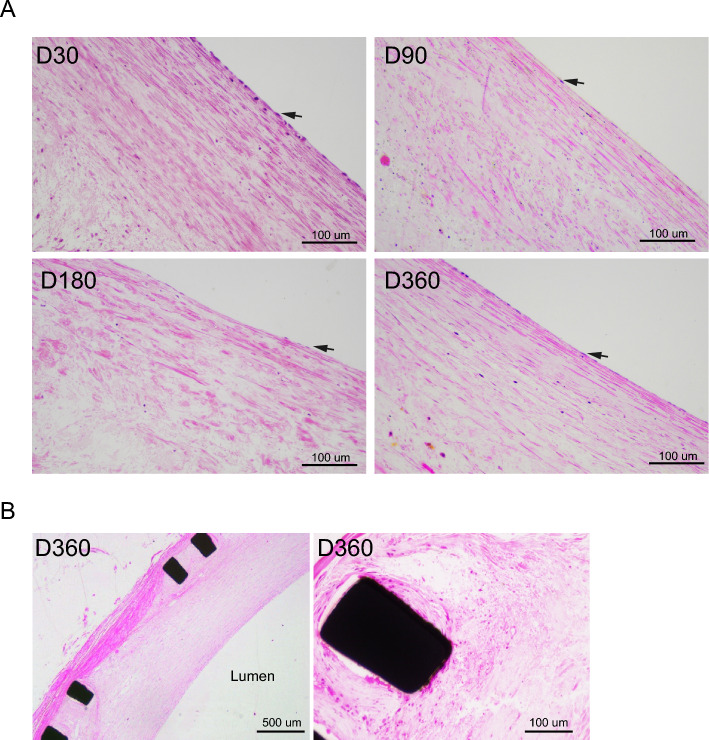
Intima hyperplasia and endothelialization. Representative H&E staining images shows the intimal hyperplasia and endothelial cell coverage at indicated timepoints post stenting. Black arrow: endothelial cells.

With HE staining of the pathological sections, the intima hyperplasia and endothelial cell coverage of the stent were observed. Both intima formation and endothelialization was seen in sections of Day-30, Day-90, Day-180 and Day-360 groups (Figs. 6 and 7). In the sections of Day-360 group, the iliac vein stent was completely covered and wrapped by hyperplastic intima (Fig. 7). The newly formed intima structure and endothelial cell coverage prevented the luminal thrombus formation. The absence of thrombosis at all follow-up time-points indirectly indicated the presence of endothelial cell coverage of the stent.

## Discussion

The overall performance of KYD stent system was satisfactory. The average lumen loss rate at day 30 after procedure was 27.3%, and at day 90, the lumen loss rate was stabilized at about 20% and decreased to 17% at day 360. Thus, these results indicated the high radial force and crush resistance of KYD stents. The inflammation was mainly seen in Day-0 and Day-30 groups. The inflammation was significantly alleviated after day 30. In addition, neither stent damage nor venous injury were found, which indicating the safety of the stents.

In recent years, more attention has been paid to IVCS as the cause of lower limb symptoms. At present, endovascular intervention technology has achieved favorable outcomes in relieving the symptoms of IVCS^13–15^. Stent treatment of iliocaval obstructions significantly decreases the clinical manifestations of chronic venous insufficiency and swelling and pain^14,16,17^.

A wide variety of dedicated venous stents were developed, such as Zilver Vena (Cook, Bjaeverskov, Denmark), Sinus Venous, Sinus Obliquus and Sinus XL Flex (Optimed, Ettlingen, Germany), Vici (Veniti; St. Louis, USA), Abre (Medtronic, Minnesota, USA) and Venovo (Bard, Tempe, USA). It is widely believed that a single “perfect” venous stent for the deep veins does not currently exist, and the type of stent used should be tailored to the needs of the specific situation^18^. For the patients with chronic iliocaval venous obstruction, especially at the iliocaval junction, one particular question for placing the currently available venous stents is whether these venous stents should extend into the inferior vena cava (IVC). One previous study reported that new thrombosis of the nonstented contralateral iliofemoral vein occurred in about 9% patients if the stents extended into IVC^19^. On the contrary, the stenosis may not be fully covered by the stents if the stents not extending into the IVC. The KYD stents with the inclined proximal end may avoid this problem.

Again, the ‘ideal’ venous stent is still not available, particularly one which performs well in the different regions of the deep venous system as each area has its particular performance needs^20^. The caliber (absolute cross-sectional area) of the iliac venous outflow controls peripheral venous pressure. Thus, the basic principle of the venous stents should mirror normal venous anatomy to adequately decompress peripheral venous hypertension^21^. The tapered KYD stents was exactly designed for fully fitting the human iliac vein anatomically (Table 8). Although we believe this is a good attempt, further study is definitely required to evaluate the advantage of tapered venous stent over cylindric one in future.

**Table 8:** Sizes of veins are expected in humans^22,23^.

Vessel segment	Diameter (mm)
Inferior vena cava	24
Common iliac vein	16–18
External iliac vein	14
Common femoral vein	12

Due to the significant difference between the distal and proximal diameters of iliac veins, tapered stent has obvious advantages, which conforms to the physiological structure of human iliac vein. Compared with the cylindrical stent, the conical stent implantation may make the changes of vascular hemodynamic much closer to the physiological condition, which can reduce the incidence of intra-stent restenosis and thrombosis^24^. It would seem logical that in the relatively immobile common iliac vein at the May–Thurner point, a stent with high radial force and crush resistance would be favorable. Conversely, in the caudal external iliac vein and common femoral vein, one could make a case for a more flexible stent as the venous segments here are more mobile^20^.

It is crucial to select proper stent size to ensure good apposition to vessel wall^25^. In current practice, slight oversizing of the stent relative to the target vessel is usually recommended^26^. Under-sizing of the stent is more harmful than slight oversizing as it may cause a permanent iatrogenic stenosis that is not easily corrected, and may also cause stent migration, even to cardiopulmonary system^27,28^. In clinical practice, the recommended stent diameter for the KYD’s iliac vein stent is 2 mm larger than the target vessel diameter. Hammer F. et al. indicated that oversizing (by 2–4 mm) of any type of stent in the venous system was important to ensure a close stent-to-vessel wall contact and avoid stent migration^29^. Consistently, Raju et al. also reported that they oversized the stent by 2 mm beyond the recommended calibre but post dilatation is restricted to the optimum outflow calibre for the segment^23^. Marston WA et al. suggested that venous stent diameter should achieve 10–30% oversizing^12^. In current study, the mean proximal and distal compression ratio of the stent was 12.4% (3.3–30.3%) and 35% (14–56.9%) (Fig. 4, Table 5) respectively. Apparently, the distal compression ratio of most KYD stents used is above the recommended value (30%). In future, redesigned KYD stents specifically for ovine iliac vein may be applied to further investigation.

In this study, each sheep was given 100 mg of oral aspirin and 75 mg of clopidogrel per day for 3 months after stenting, and then 100 mg of oral aspirin per day until being euthanized. However, the efficacy of aspirin and clopidogrel in sheep seems controversial. Spanos reported that aspirin fails to inhibit platelet aggregation in sheep because sheep platelets have an ASA resistant cyclooxygenase and may be able to aggregate by a pathway that is independent of arachidonic acid^10^. Weigand and Boos et al. reported high dosages of clopidogrel inhibited platelet aggregation merely in a low number of sheep despite sufficient absorption^11^. These studies suggested that ticagrelor and acetylsalicylic acid might be not suitable for platelet inhibition in sheep, which might provide indirect evidence to further support the performance of KYD stents with regard to venous patency. Future study is still required to evaluate the effect of ticagrelor and acetylsalicylic acid in sheep. In addition to antiplatelet therapy, there is a general consensus regarding anticoagulant therapy following venous stenting in clinical practice^30^. While Vitamin K dependent antagonists (VKAs) were the most commonly used anticoagulants in venous stenting, a major shift towards the use of direct oral anticoagulants (DOACs) have been seen in anticoagulation practices. Further study to evaluate the outcome of use of anticoagulant therapy, antiplatelet agents or combination of both following KYD stent implantation in animal model and clinical practice will be performed in future.

This study had several limitations. First, the normal iliac veins in this animal model cannot mimic the iliac vein with stenosis or obstruction that is often encountered clinically. Second, as mentioned above, KYD stent designed for human vein does not match the ovine iliac veins very well due to the anatomical difference in diameter and length between sheep and human iliac vein. Third, the weight increase of sheep may affect the results during the study. Finally, Single plane DSA is not a reliable way to measure lumen loss.

## Conclusions

The present study demonstrated the feasibility and safety of the iliac vein stent developed by KYD for implantation into the iliac vein of sheep. The iliac vein stent was easy to be manipulated and released, and there were no manipulation-related or catheter-induced adverse events, including thrombosis, air embolism, injection embolism, or death during stenting. At follow-up, there was no displacement of the stent, severe lumen loss, thrombosis, severe inflammatory response, or vessel injury. Further study is warranted to confirm the clinical efficacy and safety of KYD stents.

## Data Availability

The datasets used and/or analysed during the current study available from the corresponding author on reasonable request.
